# Evaluation of the human adaptation of influenza A/H7N9 virus in PB2 protein using human and swine respiratory tract explant cultures

**DOI:** 10.1038/srep35401

**Published:** 2016-10-14

**Authors:** Louisa L. Y. Chan, Christine T. H. Bui, Chris K. P. Mok, Mandy M. T. Ng, John M. Nicholls, J. S. Malik Peiris, Michael C. W. Chan, Renee W. Y. Chan

**Affiliations:** 1 Centre of Influenza Research and School of Public Health, LKS Faculty of Medicine, The University of Hong Kong, Hong Kong SAR, China; 2 The HKU-Pasteur Research Pole, School of Public Health, LKS Faculty of Medicine, The University of Hong Kong, Hong Kong SAR, China; 3 Department of Pathology, LKS Faculty of Medicine, The University of Hong Kong, Queen Mary Hospital, Hong Kong SAR, China; 4 Department of Paediatrics, Faculty of Medicine, The Chinese University of Hong Kong, Hong Kong SAR, China

## Abstract

Novel avian H7N9 virus emerged in China in 2013 resulting in a case fatality rate of around 39% and continues to pose zoonotic and pandemic risk. Amino acid substitutions in PB2 protein were shown to influence the pathogenicity and transmissibility of H7N9 following experimental infection of ferrets and mice. In this study, we evaluated the role of amino acid substitution PB2-627K or compensatory changes at PB2-591K and PB2-701N, on the tropism and replication competence of H7N9 viruses for human and swine respiratory tracts using *ex vivo* organ explant cultures. Recombinant viruses of A/Shanghai/2/2013 (rgH7N9) and its mutants with PB2-K627E, PB2-K627E + Q591K and PB2-K627E + D701N were generated by plasmid-based reverse genetics. PB2-E627K was essential for efficient replication of rgH7N9 in *ex vivo* cultures of human and swine respiratory tracts. Mutant rgPB2-K627E + D701N replicated better than rgPB2-K627E in human lung but not as well as rgH7N9 virus. The rgPB2-K627E mutant failed to replicate in human type I-like pneumocytes (ATI) and peripheral blood monocyte-derived macrophages (PMϕ) at 37 °C while the compensatory mutant rgPB2-K627E + Q591K and rgPB2-K627E + D701N had partly restored replication competence in PMϕ. Our results demonstrate that PB2-E627K was important for efficient replication of influenza H7N9 in both human and swine respiratory tracts.

In March 2013, a novel avian-origin H7N9 virus emerged in China. As of 20^th^ July 2016, a total of 795 laboratory confirmed human infections and 314 deaths were reported from 19 provinces and municipalities in Mainland China, Hong Kong, Macau, Taiwan, Malaysia and Canada. H7N9 viruses have spread from Eastern China in the first wave of the outbreak in early 2013, to Southern China in the second wave, and has now become enzootic in multiple provinces in China. Since infection in poultry is asymptomatic, H7N9 virus is likely to spill over borders and spread across the region in a pattern similar to that observed with H5N1 and H9N2 influenza viruses previously^1^,^2^. Human H7N9 infections can lead to a rapidly progressing viral pneumonia, acute respiratory distress syndrome (ARDS) and multi-organ failure^3^, especially in older patients and in those with underlying co-morbidities.

Most zoonotic H7N9 disease is associated with exposure to poultry within live poultry markets^4^,^5^ with no evidence of sustained human-to-human transmission. Active surveillance of chickens in live poultry markets in five provinces in China showed an average isolation rate of 3.0%^1^. Phylogenetic analysis indicated that the novel H7N9 virus originated through reassortant of avian influenza viruses from wild aquatic birds and poultry; the hemagglutinin gene and the neuraminidase gene respectively, being derived from H7N3 and H7N9 viruses in domestic ducks while the six “internal” genes were derived from avian H9N2 viruses found in poultry in Eastern Asia^4^,^6^,^7^. These viruses have acquired multiple mammalian adaptations. The HA protein possesses alanine (A) at position 160 and leucine (L) at position 226 (H3 numbering) which is expected to enhance the receptor binding specificity to mammalian α-2,6 sialic acid receptors, enabling the virus cross species from birds to humans^8^,^9^. Deletion of amino acids in the stalk region of the NA protein (at position 69 to 73) was previously found in HPAI H5N1 virus and is an adaptation of influenza viruses to replication in terrestrial poultry such as chicken and may also affect viral replication efficiency and tissue tropism in the respiratory tract^4^. The polymerase basic protein 2 (PB2) of avian influenza viruses typically has glutamic acid (E) at position 627 while lysine (K) was usually seen in viruses circulating in humans^10^. While H7N9 viruses isolated from poultry have PB2-627E, some viruses isolated from humans have a PB2-E627K amino acid substitution, which is known to enhance the viral replication efficiency and increase virulence in mice^10^,^11^,^12^. The PB2-627K mutation has been shown to enhance polymerase activity in human cells and increase pathogenicity in mice^13^,^14^,^15^. Some of the human H7N9 isolates retained PB2-627E and gained other compensatory mammalian markers such as PB2-Q591K and D701N^16^. Adaptive mutations Q591K and D701N in PB2 were associated with increased polymerase activity in mammalian cells^17^,^18^,^19^. Direct evidence of the role of these “mammalian adaptations” in humans is lacking.

We previously demonstrated that the human influenza H7N9 isolates (A/Shanghai/1/2013 and A/Shanghai/2/2013) replicate more extensively and more efficiently in *ex vivo* cultures of human bronchus and lung than does H5N1 viruses^9^. However, in comparison to H5N1 viruses which are potent inducers of pro-inflammatory cytokine responses in primary human cells, the human H7N9 viruses were moderate inducers of such cytokines. Similar studies have been conducted using swine respiratory tissue explant cultures to address the susceptibility of the swine respiratory tract to H7N9 viruses^20^. This is relevant since swine have long been recognized as a potential “mixing vessel” of human and avian influenza viruses^21^,^22^, together with its critical role in the emergence of pandemic H1N1 in 2009^23^. Experimental infection of pigs has led to productive viral replication but no transmission from pig to pig^24^. However, given the close contact between pigs, poultry and humans in agricultural settings in China, it is important to investigate the impact of genetic adaptations of H7N9 viruses in swine.

In this study, we used recombinant A/Shanghai/2/2013 (rgH7N9) to investigate the importance of PB2-E627K mutation and related compensatory mutations in PB2, namely Q591K and D701N, on tissue tropism, replication competence and cytokine induction in *ex vivo* explant cultures of human and swine respiratory tract.

## Results

### Replication kinetics of rgH7N9 virus and rgH7N9-PB2 mutants in DF-1 cells and MDCK cells at 33 °C and 37 °C

**Figure 1:**
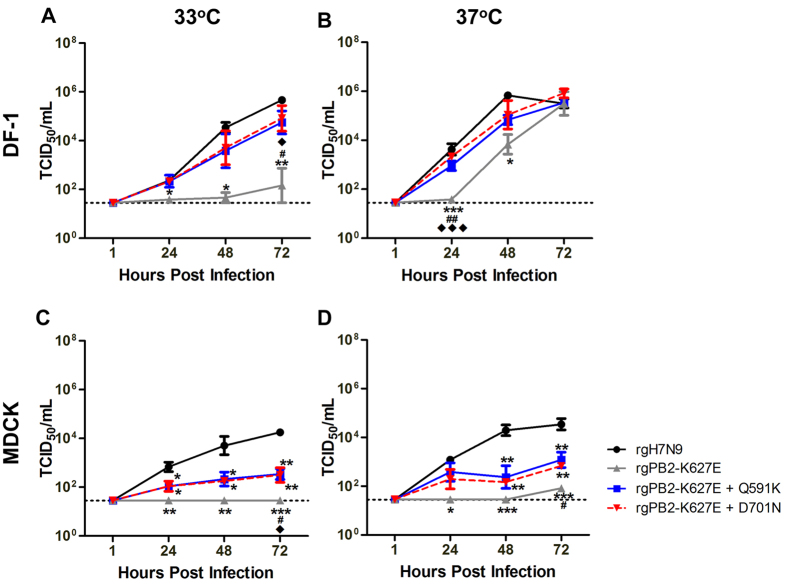
Replication kinetics of PB2 mutant viruses in DF-1 (chicken fibroblast) and MDCK (canine kidney epithelial cell). Viral replication kinetics of HA mutants of H7N9 viruses in DF-1 (**A**,**B**) and MDCK (**C**,**D**) cells infected at a MOI of 0.01 at 33 °C (**A**,**C**) or 37 °C (**B**,**D**). Bar charts show the mean virus titre pooled from at least three independent experiments. The horizontal dotted line denotes the limit of detection in the TCID_50_ assay. Error bars show SEM. *p < 0.05, **p < 0.01, ***p < 0.005, rgH7N9 vs all PB2 mutants; #*p* < 0.05, ##*p* < 0.01, rgPB2-K627E + Q591K vs rgPB2-K627E; ^♦^*p* < 0.05, ^♦♦♦^*p* < 0.005, rgPB2-K627E + D701N vs rgPB2-K627E.

The replication kinetics of the rgH7N9 and its PB2 mutants in an avian chicken fibroblast cell line (DF-1) and a mammalian cell line (MDCK) at 33 °C and 37 °C were compared (Fig. 1). In the avian DF-1 cells, rgPB2-K627E demonstrated its temperature sensitive phenotype and replicated poorly at 33 °C (Fig. 1A) while the mutant viruses with the human compensatory markers, rgPB2-K627E + Q591K and rgPB2-K627E + D701N were able to replicate to similar titers as rgH7N9 which has PB2-627K (Fig. 1A). At 37 °C, all H7N9 viruses achieved similar viral titers by 72 hpi indicating their replication competence in avian cells, though rgPB2-K627E virus had a delayed replication kinetics with a lower viral titer at 24 hpi (Fig. 1B).

In the mammalian MDCK cells, the replication kinetics of rgH7N9 (PB2-627K) was similar at both 33 °C and 37 °C with greater replication efficiency than all three mutants with PB2-627E (Fig. 1C,D). The mutant virus rgPB2-K627E failed to replicate at 33 °C and yielded limited virus progeny at 37 °C at 72 hpi. PB2-K627E mutants with compensatory human adaptation markers, PB2-Q591K or D701N, had partially restored the replication competence in MDCK cells at both temperatures, but they failed to replicate as efficiently as rgH7N9 which possesses PB2-627K.

### Replication competence of rgH7N9 and PB2 mutants in *ex vivo* cultures of human bronchus and lung at 37 °C

**Figure 2:**
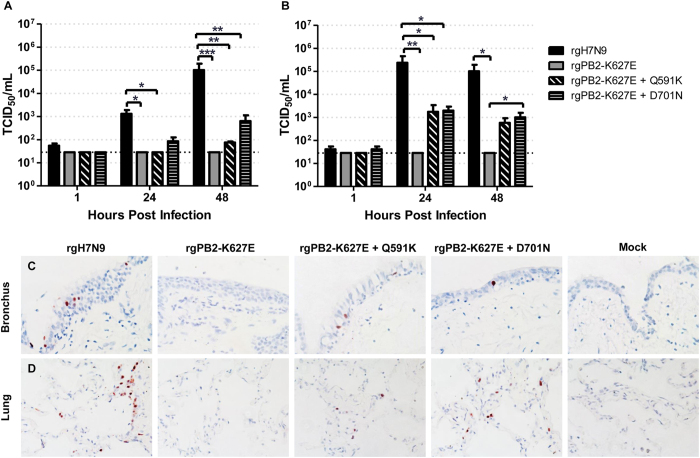
Viral replication kinetics and Tissue tropism of rgH7N9 and its PB2-mutant viruses in *ex vivo* cultures of human respiratory organs. Human bronchus (**A**) and lung (**B**) were infected with 10^6^ TCID_50_/mL of influenza viruses at 37 °C. Bar charts show the mean virus titre pooled from at least three independent experiments. The horizontal dotted line denotes the limit of detection in the TCID_50_ assay.; error bars show SEM. Key: TCID_50_ = tissue culture infective dose. *p < 0·05, **p < 0.01, ***p < 0.005. Formalin-fixed paraffin-embedded sections of human bronchus (**C**) and lung (**D**) after 24 h infection with rgH7N9, rgPB2-K627E, rgPB2-K627E + Q591K and rgPB2-K627E + D701N viruses and mock. Sections were stained with a monoclonal antibody against the influenza nucleoprotein with positive cells identified as a red-brown colour and arrows indicated the infected cell. Magnification, ×400.

The rgH7N9 virus replicated to significantly higher titers than each of the PB2 mutants in the human bronchus (Fig. 2A). It also had a trend for a more efficient replication than each of the PB2 mutants in the lung (Fig. 2B) at all time-points. The rgPB2-K627E mutant virus failed to replicate in either human bronchus or lung. The introduction of compensatory human markers, PB2-Q591K and D701N, partly rescued the replication competence of the rgPB2-K627E virus in lung. The mutant virus with rgPB2-K627E + D701N showed partly restored replication competence in bronchus but its counterpart virus, rgPB2-K627E + Q591K did not.

### Tissue tropism of the rgH7N9 and PB2 mutants in *ex vivo* cultures of human bronchus and lung

Immunohistochemical staining of the infected tissues indicated that rgH7N9 virus possessing PB2-627K infected the human bronchial epithelium and the alveoli more extensively than its PB2 mutants (Fig. 2C,D). The rgPB2-K627E mutant virus failed to replicate in the *ex vivo* cultures of human respiratory epithelia (Fig. 2C,D). Moreover, the extent of infection correlated well with the viral replication titers. There were very limited influenza antigen positive cells in the rgPB2-K627E + Q591K and rgPB2-K627E + D701N inoculated *ex vivo* culture of human bronchus (Fig. 2C) while more infected cells were shown in these mutant viruses infected lung tissues (Fig. 2D).

### Replication competence and tissue tropism of rgH7N9 and PB2 mutants in *ex vivo* cultures of swine trachea, bronchus and lung

**Figure 3:**
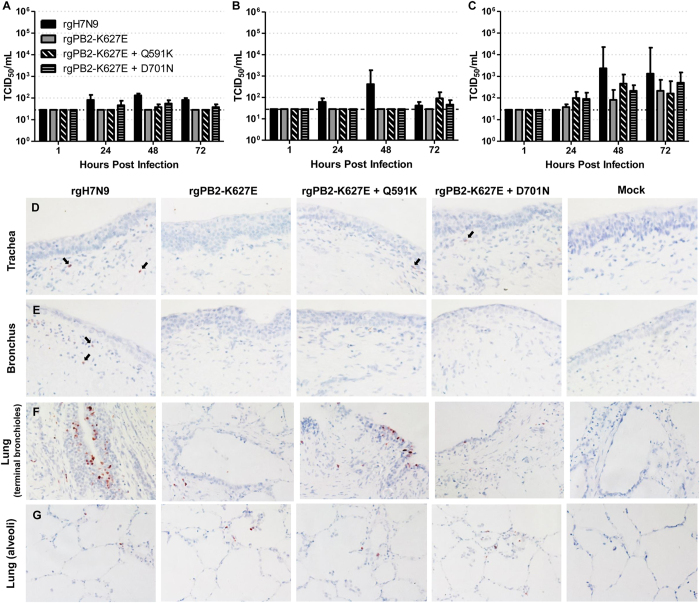
Viral replication kinetics and Tissue tropism of influenza rgH7N9 viruses in *ex vivo* cultures of swine respiratory organs. Swine trachea (**A**), bronchus (**B**) and lung (**C**) were infected with 10^6^ TCID_50_/mL of influenza viruses at 37 °C. Bar charts show the mean virus titre pooled from at least three independent experiments. The horizontal dotted line denotes the limit of detection in the TCID_50_ assay.; error bars show SEM. Key: *p < 0·05, ** *p* < 0.01, *** *p* < 0.005. Formalin-fixed paraffin-embedded sections of swine trachea (**D**), bronchus (**E**), terminal bronchioles (**F**) and alveoli (**G**) in lung after 24 h infection with rgH7N9, rgPB2-K627E, rgPB2-K627E + Q591K and rgPB2-K627E + D701N viruses and mock. Sections were stained with a monoclonal antibody against the influenza nucleoprotein with positive cells identified as a red-brown colour and arrows indicated the infected cell. Magnification, ×400

rgH7N9 virus failed to replicate in *ex vivo* cultures of swine trachea but replicated in bronchus and lung (Fig. 3A–C). The mutant viruses rgPB2-K627E + Q591K and rgPB2-K627E + D701N also replicated in *ex vivo* lung cultures (Fig. 3C) while minimal or no viral replication was observed in swine trachea and bronchus (Fig. 3A,B). rgH7N9 and all PB2 mutants replicated to lower titers than H1N1pdm did, especially in the swine lung (Supplementary Fig. 1A).

In *ex vivo* culture of the swine respiratory tract, the epithelial cells in the terminal bronchioles were the main target cell types. The most extensive influenza nucleoprotein antigen positive cells were seen with the rgH7N9 virus, followed by rgPB2-K627E + Q591K mutant virus-infected tissues (Fig. 3F). Limited numbers of infected alveolar macrophages and type II pneumocytes were seen in the virus inoculated swine alveolar *ex vivo* cultures (Fig. 3G). None of the viruses were able to infect the *ex vivo* cultures of swine trachea and bronchus, with the exception of some positive cells found in the interstitial tissue within the trachea (Fig. 3D) and bronchus (Fig. 3E). H1N1pdm also failed to infect the swine trachea and bronchus, the extent of infection in the terminal bronchioles and alveoli in the swine lung was similar to that of rgH7N9 (Supplementary Fig. 1B–E).

### Replication kinetics of rgH7N9 and PB2 mutant viruses in human type I-like cells and human peripheral blood derived macrophages

**Figure 4:**
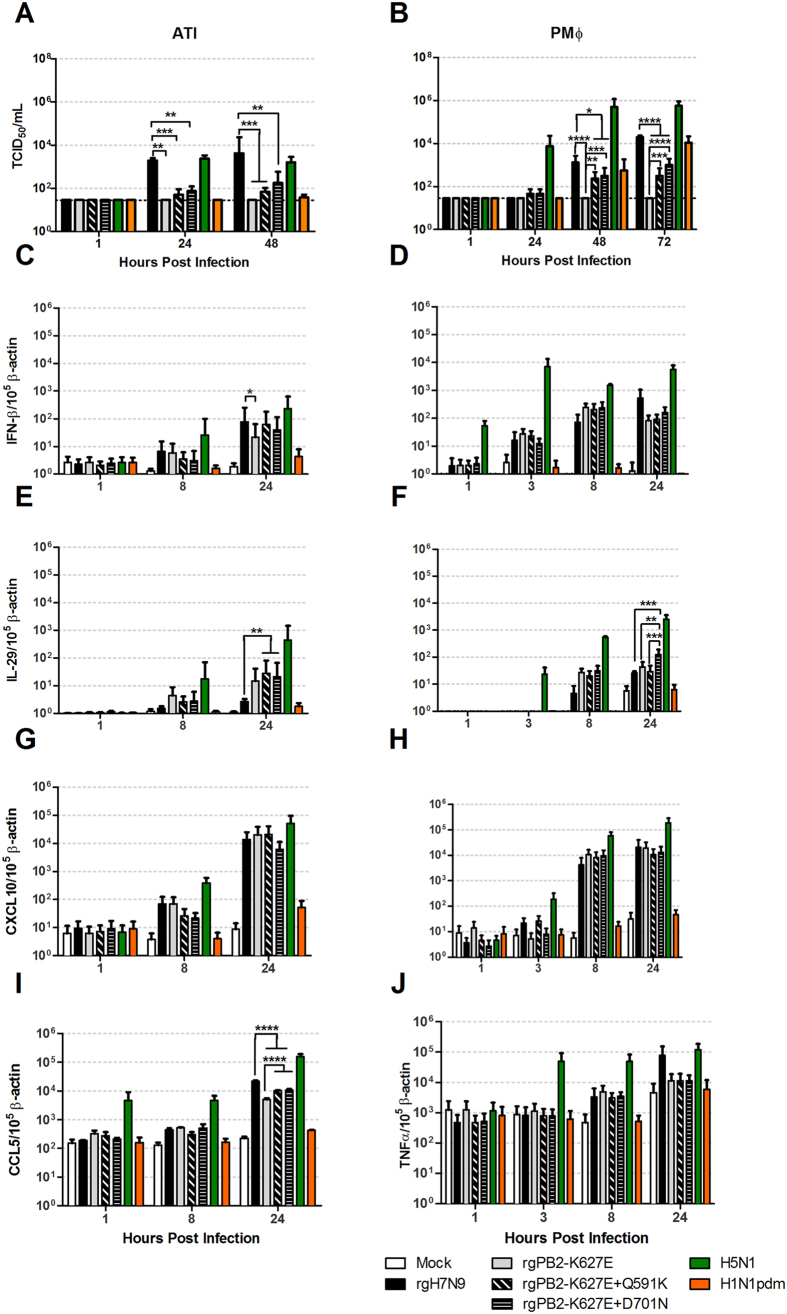
Replication kinetics, cytokine and chemokine mRNA expression profile in type I-like pneumocytes (ATI) and peripheral blood monocyte-derived macrophages (PMϕ). Viral replication kinetics of PB2 mutants of H7N9 viruses in ATI (**A**) and PMϕ (**B**) cells infected at a MOI of 0.01 at 37 °C. Human primary cell infected with mock, rgH7N9 and PB2 mutant viruses, H5N1, and H1N1pdm viruses at a MOI of 2 at 37 °C. Expression of IFN-β (**C**,**D**), IL29 (**E**,**F**), CXCL10 (**G**,**H**) and CCL5 (**I**) and TNFα (**J**) at 1, 8, 24 hpi in ATI and 1, 3, 8, 24 hpi in PMϕ. Graphs show mean mRNA copies expressed per 10^5^ β-actin copies from three independent experiments; error bars show SEM. Two-way ANOVA followed by *Bonferroni’s* multiple comparison test were performed to compare rgH7N9 and its mutants. *p < 0.05, **p < 0.01, ***p < 0.005, ****p < 0.0001.

With a MOI of 0.01 infection, rgH7N9 replicated to titers significantly higher than all its PB2 mutants at all the time points examined in both type I-like pneumocytes (ATI) and peripheral blood monocyte-derived macrophages (PMϕ) (Fig. 4A,B). rgPB2-K627E failed to replicate in both primary cells, while compensatory human markers PB2-Q591K and D701N partially restored the viral replication efficiency.

Therefore, we investigated if the lack of replication was due to the lack of infectivity or the incompetence in producing new progeny viruses. We compared the infection rate of these viruses in ATI at 24 hpi (Supplementary Fig. 2) and PMϕ at 8 hpi (Supplementary Fig. 3). With a MOI of 2 infection, there were no statistical significant differences in the infection rate among the rgH7N9 virus to its mutants, ranging from 61–75% in ATI (Supplementary Fig. 2E) and 80–91% in PMϕ.

### Cytokine and chemokine gene expression in the H7N9 virus infected human ATI and PMϕ

The cytokine and chemokine gene expression was compared among the rgH7N9 and its mutants in ATI and PMΦ at a MOI of 2. In ATI, rgPB2-K627E + Q591K and rgPB2-K627E + D701N induced significantly more IL-29 gene expression than the rgH7N9 (Fig. 4E) while mutant rgPB2-K627E induced significantly lower IFN-β and CCL5 than rgH7N9 (Fig. 4C,I) at 24 hpi. In PMϕ, though the cytokine and chemokine expression induced by the PB2 mutants was comparable with rgH7N9, rgPB2-K627E + D701N upregulated the expression of IL-29 compared to the other H7N9 viruses (Fig. 4F). In accordance with our previous findings^9^, H7N9 virus represented by rgH7N9 in this study, were found to be moderate inducers of pro-inflammatory cytokine and chemokine with H5N1 being the high cytokine-inducer and H1N1pdm being a low cytokine-inducer for IFN-β (Fig. 4C,D), IL-29 (Fig. 4E,F) and CXCL10 (Fig. 4G,H) in both ATI and PMϕ, CCL5 in ATI (Fig. 4I) and TNF-α (Fig. 4J) in PMϕ.

## Discussion

In this study, we evaluated the effects of PB2-E627K mutation as well as two mammalian adaptation markers, PB2-Q591K and D701N on H7N9 virus replication competence in *ex vivo* cultures of human and swine respiratory tracts and *in vitro* cultures of human primary ATI and PMϕ. While avian H7N9 viruses invariably have PB2-627E, A/Shanghai/2/2013 (Sh2) (H7N9) virus which was isolated from a patient with severe disease had acquired the mammalian adaptation mutation PB2-E627K, and this is reflected in recombinant rgH7N9 virus used in this study. Tissue tropism and virus replication competence of rgH7N9 virus and its PB2 mutants were compared. Recombinant H7N9 virus with PB2-627K replicated to similar titers with H1N1pdm in *ex vivo* cultures of human bronchus and lung as we previously described^9^. We demonstrated that H7N9 possessing PB2-627E failed to infect and replicate in the *ex vivo* cultures of human bronchus, lung, *in vitro* cultures of human ATI and PMϕ and in *ex vivo* cultures of swine trachea and bronchus suggesting that the avian virus is not adapted for replication in the human or swine respiratory tract. The compensatory mammalian adaptation mutations in PB2, Q591K or D701N could partially rescued the virus replication in human and swine cells and tissues though it was not reaching the full competence of the rgH7N9 virus with the PB2-627K. This emphasized the key role of PB2-E627K in contributing to the efficient viral replication of H7N9 in the human respiratory tract. These findings are in agreement with others who have found that lysine in position 627 in PB2 was found to be essential for mammalian adaptation in terms of the enhancement of polymerase activity, viral growth kinetics and virulence in mice when compared with the mutants of Sh2 or A/Anhui/1/2013 (H7N9) having PB2-627E^13^,^14^.

From our observation, the viral replication efficiency of H7N9 and the PB2 mutants in *ex vivo* cultures of human respiratory tract was greater than in swine respiratory tract for approximately 2-fold difference in viral titer. This might contribute to the differential polymerase activities in human cells and porcine cells^25^, the relatively lower polymerase activity detected in porcine cells may be responsible for the overall low viral titers resulted from H7N9 virus infection in the *ex vivo* cultures of swine respiratory tissues^26^,^27^,^28^. The profiles of sialic acid distribution in human and swine respiratory tract might play a crucial role as well though the distribution pattern of sialic acid receptors in swine and human respiratory tracts are similar^27^,^29^,^30^, but higher Galα1-3Gal expression and comparatively rare extended sialylated LacNAc repeats can be observed in swine^31^.

Several previous studies have investigated the replication and infection potential of H7N9 using swine as experimental model, both *in vivo* and *ex vivo*. Here, we isolated the *ex vivo* cultures of swine trachea, bronchus and lung from intact respiratory organs of 6-month old pigs (*Sus scrofa domestica*). These pigs were farm-raised, exposed to field conditions and represent the natural population of animals^31^. Jones *et al*., in contrast, used the explant cultures of tracheal and lung explants of one-week-old laboratory piglets to examine the infectivity of H7N9 in swine. They found that three human H7N9 viruses including A/Anhui/1/2013, A/Shanghai/1/2013 and A/Shanghai/2/2013 replicated efficiently in trachea and lung^20^. In our case, although no replication and positive staining were found in the swine trachea after virus inoculation, the influenza nucleoprotein antigen positive cells were also found in terminal bronchiolar epithelial cells in the lung. The lectin binding and glycan array profiles of the respiratory tracts of adult domestic pigs are different from infant pigs, the binding of *Sambucus nigra* agglutinin (SNA, a lectin binds α-2,6 glycans) was strong for the upper respiratory tract of both infant and adult pigs but binding to *Maackia amurensis* agglutinin-I (MAA-I, a lectin binds α-2,3 N-glycans) and MAA-II (a lectin binds α-2,3 O-glycans) was only observed infant pigs. There was a strong binding of SNA lectin to the alveoli of infant pigs but weak to that of adult pigs^27^,^31^. Therefore, it is possible that age of pigs may affect the permissiveness for H7N9 virus replication. This can lead to variations in the determination of virus tropism, infection and transmission.

Our data in swine *ex vivo* cultures is compatible with previous data from pigs experimentally infected with H7N9 where the viral RNA load in the trachea and bronchus was low^24^. In addition, Zhu *et al*. demonstrated by immunohistochemistry that H7N9 virus also targeted the nasal turbinates, however we have not examined the nasal turbinates in the current study. Liu *et al*. infected young domestic pigs *in vivo* with A/Anhui/1/2013 recombinant viruses^32^. They found that the wild type and 627E viruses replicated well in the lung and trachea and was found transmissible between direct contact animals^32^. Our observations suggest that H7N9 with either PB2-627K or E is able to infect and replicate the *ex vivo* cultures of swine lung and thus providing a virus reservoir for possible transmissions, however, the lack of efficient replication of H7N9 in swine conducting airways, the trachea and bronchus, would imply that transmission could be inefficient. The tropism observations presented in this paper partly addressed the reason why there is not yet a report on the isolation of H7N9 from pigs in the field^33^,^34^,^35^.

Apart from the study in pig models, the effect of amino acid substitution in PB2 position 627 of H7N9 were also evaluated in chickens and ferrets. A recent report suggested that an avian H7N9 virus isolated from pooled oropharyngeal swabs of Silkie chickens (A/SCk/HK/1772/2014) with PB2-627E transmitted efficiently among chickens via direct contact. The transmission efficiency of this isolate was comparable to that found in a human H7N9 isolate with PB2-627V/E/K polymorphism (A/HK/3263/2014)^36^. When a chicken-to-ferret transmission experiment was performed using this Silkie chicken strain, a rapid increase in the proportion of 627K over 627E was observed in the nasal washes of transmitted ferrets. The E627K adaption in PB2 gene was therefore shown to be associated with mammalian adaptation in ferrets^5^,^37^,^38^. Besides, we showed that in contrast to the tropism data found in the tissue and cells of human and swine, rgPB2-K627E could still replicate in the chicken fibroblast DF-1 cells at 37 °C, albeit less efficiently than rgH7N9 or the mutants rgPB2-K627E + Q591K or rgPB2-K627E + D701N. The rgPB2-K627E virus replicated poorly if at all in DF-1 or MDCK cells at 33 °C, exhibiting its temperature sensitive phenotype^39^,^40^.

Apart from the determination of tissue tropism, the effect of PB2 mutations in H7N9 virus was also evaluated. Our results demonstrated that, with a similar percentage of infection, the proinflammatory cytokines and chemokines induced by the rgH7N9 and its mutants in ATI and PMϕ was generally similar, except IL-29 gene was upregulated by rgPB2-K627E + Q591K and rgPB2-K627E + D701N in ATI and by rgPB2-K627E + D701N in PMΦ compared to rgH7N9 at 24 hpi. The reduced mRNA expression levels of IFN-β and CCL5 in ATI infected with rgPB2-K627E in comparison to rgH7N9 with PB2-627K followed the similar story in a mouse study that H7N9 with PB2-627K would induce a significantly higher level of proinflammatory in the lungs of mice than those infected with rgPB2-627E^13^. In general, the overall profile of cytokine and chemokine induction by H7N9 virus, follow the previous findings that H7N9 induced a lower cytokine response when compared to HPAI H5N1 as shown in human primary cultures^9^ and mice^41^.

Although rgPB2-K627E viruses failed to replicate in human ATI and PMϕ cells in the low MOI experiment, these cells were infected at similar rate and cytokine responses were elicited in the infection with a MOI of 2. Influenza viruses are detected through the recognition of toll-like receptor (TLR) 3 expressed in the endosome and on the surface of human respiratory epithelial cells^42^,^43^,^44^ and in the phagosome of macrophages^45^,^46^,^47^. Thus abortive infection may be sufficient to trigger cytokine responses. Infected cells are sensed by TLR3 with the presence of double-stranded RNA (dsRNA) and leads to the production of pro-inflammatory cytokines dependent on the expression of nuclear factor-κB (NF-κB), type I interferon and IFN-stimulated genes (ISGs)^45^,^48^,^49^. Macrophages are active innate immune cells able to patrol around to detect potential pathogens by amoeboid movement. One of the characteristic differences between macrophages and lung epithelial cells is the function of phagocytosis for the removal of infected or dying cells^50^,^51^. Although rgPB2-K627E was not replicating in PMϕ, it might be possible that PMϕ phagocytoses the influenza virus-infected and apoptotic cells therefore activates the induction of pro-inflammatory cytokines since PMϕ is capable of secreting cytokines once they exposed to any inflammatory stimuli^50^,^52^,^53^.

To conclude, rgH7N9 possessing PB2-K627E failed to replicate and infect in both *ex vivo* cultures of human bronchus and lung and in *in vitro* model of human ATI and PMϕ. Thus, PB2-E627K, or the compensatory adaptations such as PB2-Q591K or D701N would be essential to at least partly rescue the replication competence of virus possessing PB2-627E in the mammalian system. The current *ex vivo* cultures of human and swine lung, and *in vitro* culture of primary human peripheral blood derived macrophages would be a good risk assessing model for the virus competence in replication and host innate immune response induction. Our experimental findings explain why many H7N9 virus isolates from humans have one or other of these mammalian adaptation mutations in PB2.

## Methods

### Viruses

Table 1 listed the viruses used in this study. All the recombinant H7N9 viruses were generated by plasmid based reverse genetics of influenza virus A/Shanghai/2/2013^13^. Virus stocks used for these experiments were propagated and titrated in the Madin-Darby canine kidney (MDCK) cells to determine the tissue culture infection dose 50% (TCID_50_). MDCK cells were cultured in Eagle’s minimal essential medium (MEM) containing 25 mM HEPES, 10% fetal calf serum (FBS) and 100 U/ml penicillin and 100 μg/ml streptomycin. Chicken fibroblast DF-1 cells were cultured in Dulbecco’s modified Eagle’s medium (DMEM) containing 10%FBS and 1%PS.

### TCID_50_ assay

Sub-confluent MDCK 96-well tissue culture plates were prepared one day before the virus titration assay. Cells were washed once with warm phosphate-buffered saline (PBS) and replenished with serum-free MEM with 1%PS and 2 μg/ml of tosylsulfonyl phenylalanylchloromethyl ketone (TPCK)–treated trypsin. Serial dilutions of culture supernatants from experiments, ranging from 0.5log to 7log, were performed before adding the virus dilutions onto the plates in quadruplicate and cytopathic effect (CPE) was monitored daily. The end point of viral dilution leading to CPE in 50% of inoculated wells was estimated using the Karber method.

### *Ex vivo* organ cultures of human respiratory tract

Fresh biopsies of human normal bronchus and lung tissues were obtained from patients undergoing surgical resection of bronchus and lung tissues at Queen Mary Hospital. Informed consent has been obtained from all subjects. This study was approved by the Institutional Review Board of the University of Hong Kong and Hospital Authority Hong Kong West Cluster (UW 14-119) and all methods involving human tissues were performed in accordance with relevant guidelines and regulations. Tissue fragments of bronchial tissue were placed on a sterile surgical sponge for air-liquid interface (ALI), while lung tissue were cut into thin slices and cultured with F12K medium in 24-well plate as previously described^9^,^54^. These *ex vivo* cultures were maintained at 37 °C for culture and infection.

### *Ex vivo* organ cultures of swine respiratory tract

Intact respiratory organs were obtained from freshly slaughtered pigs in Hong Kong Sheung Shui slaughterhouse and delivered within 3 hours in a sterile plastic box at 4 °C. A tracheal swab was taken to exclude current influenza infection in the animal. It was tested for influenza M gene by real-time qPCR and data generated from any swine tissues positive for influenza A virus in the swab sample were excluded. Tracheobronchial epithelium (TBE) was prepared by removing the mucosa and submucosa from the cartilage with a pair of fine forceps and a sharp scalpel. Lung slices were prepared by perfusing the lung with cold transport medium and then 1% agarose in PBS using a clear tracheal tube 4.0 mm through the bronchiole until the lobe was fully expanded. Lung tissue was cut in cubes and embedded with 4% agarose. The agarose-embedded lung tissue was cut in slices, using a cryotome blade with <1 mm thickness. TBE epithelia and lung slices were cut into circular snippets with 5 mm diameter using a disposable biopsy punch (Miltex). These thin snippets were placed on surgical sponge in a 12-well culture plate. 1.5 ml corresponding medium was added into each well with the epithelial explants and the surgical sponge floating on the medium, as previously described^31^. Tracheal epithelium was cultured in a 1:1 mixture of RPMI 1640 and DMEM high glucose with 1%PS, 1 μg/ml gentamicin, 0.3 mg/ml glutamine, bronchial epithelium was cultured in MEM with 1%PS, 1 μg/ml kanamycin, 0.3 mg/ml L-glutamine and 20 mM HEPES while the thin lung slices were cultured in DMEM high glucose with 2.5 μg/ml bovine insulin, 0.5 μg/ml hydrocortisone, 0.5 μg/ml Vitamin A (Sigma, USA) and 0.1 mg/ml gentamycin. Culture medium of these explants was changed every hour within the first four hours and incubated in a 37 °C water-jacketed incubator with 5% CO_2_. All experiments involving swine tissues were carried out in accordance with relevant guidelines and regulations. All experimental protocols were approved by the Institutional Review Board of the University of Hong Kong.

### Infection of *ex vivo* cultures of human and swine respiratory tract

The respiratory explant cultures were inoculated with influenza viruses with comparable infecting virus dose of 10^6^ TCID_50_/ml, by submerging the explants tissue in 1 ml of virus dilution for 1 h at 37 °C. Tissues were washed 3 times with 5 ml PBS to remove any unbounded virus. The supernatant of the infected culture was collected for virus titration to study the kinetics of virus replication at 1, 24, 48 and 72 h post infection (hpi).

### Immunohistochemical staining for influenza A virus antigen

To examine the infectivity of the viruses, the control and infected respiratory tissues were collected at 24, 48 and 72 hpi and fixed in 10% neutral buffered formalin and processed for paraffin embedding. Immunohistochemistry using a mouse anti-influenza nucleoprotein antibody (HB65, EVL Laboratories, Netherlands) was performed to examine the IAV antigen as previously described^54^.

### Culture of human type I-like pneumocytes (ATI)

After the removal of visible bronchi, the lung tissue was minced into pieces of thickness <0.5 mm with scissors. Lung pieces were washed with Hanks’s balanced salt solution at pH7.4 for three times to remove macrophages and blood cells. A combination of 0.5% trypsin and 4 U/ml elastase (Worthington Biochemical Corporation, USA) was added to the lung pieces and incubated for 40 min in a 37 °C water bath with shaking for digestion and stopped with DMEM/F12 medium with 40%FBS and DNase I (350 U/ml) (Sigma, USA). Undigested lung fragments were separated with a 50 μm pore size disposable cell strainer and cell clumps were dispersed by repeated pipetting for 10 min. Single cell suspension in flow-through was centrifuged and resuspended in a 1:1 mixture of DMEM/F12 medium and small airway growth medium (SAGM) (Lonza, USA) with 5% FBS and 350 U/ml DNase I. Resuspended cells were plated on a tissue culture flask (Corning, USA) for 90 min adhesion at 37 °C. The non-adherent cells were centrifuged, pelleted and resuspended with SAGM with supplements and 1%PS and plated on a new culture flask. The growth medium was changed daily starting from 60 h after cell plating. At 75% confluence, the ATI cell layer was trypsinised for seeding.

### Human peripheral blood monocyte-derived macrophages (PMϕ)

Buffy coats of healthy blood donors were obtained from the Hong Kong Red Cross Blood Transfusion Service and peripheral blood leucocytes were separated by centrifugation on a Ficoll-Paque density gradient and purification of monocytes were done by adhering on plastic petri dishes as previously described^55^. Monocytes were seeded onto tissue culture plates in RPMI 1640 medium supplemented with 5% heat-inactivated autologous plasma. Immunostaining for surface receptor CD14 (BD Biosciences, USA) was done to ensure the purity of monocyte preparations. A 14-day differentiation was done and the culture medium was changed to macrophage serum free medium SFM (Gibco, USA) a week before infection.

### Virus infection of primary cell cultures *in vitro*

Human ATI and PMϕ were infected with influenza A viruses at a multiplicity of infection (MOI) of two for the analysis of cytokine and chemokine expression or at MOI of 0.01 for viral replication kinetics. Serum-free MEM with 1%PS was used as inoculum for mock treatment. The cell cultures were incubated with the virus inoculum for 1 h in a water-jacketed 37 °C incubator with 5% CO_2_. The cells were rinsed three times with warm PBS and replenished with the appropriate growth medium. 130 μl of culture supernatant was collected from each treatment at 1, 24 and 48 hpi for ATI and 1, 24, 48 and 7 2hpi for PMϕ for virus replication kinetics study. The infected cells were harvested for mRNA collection at 1, 8 and 24 hpi for ATI and 1, 3, 8 and 24 hpi for PMϕ. 4% paraformaldehyde (PFA) fixed cell monolayer seeded on coverslips were collected at 24 hpi for ATI and 8 hpi for PMϕ.

### Quantification of pro-inflammatory cytokine and chemokine mRNAs by quantitative RT-PCR

Human ATI and PMϕ were lysed in 350 μl RLT buffer with beta-mercaptoethanol after the infection in MOI of 2. RNA extraction was performed using an RNeasy minikit (Qiagen, Germany) following the manufacturer’s protocol with DNase treatment followed by reverse transcription using PrimerScript RT reagent Kit (Takara, China). The gene expression level was quantified by real-time PCR amplification using ViiA™ 7 Real-Time PCR System (Applied Biosystem, USA). Gene expression profiles of cytokines tumor necrosis factor alpha (TNF-α), interleukin (IL)-29, interferon β (IFN-β); chemokines CXCL10, and CCL5 were normalized using housekeeping gene β-actin mRNA at the time points stated above. Absolute copy numbers of cytokine, chemokine and β-actin gene were determined from a standard curve generated from a standard plasmid with a known copy number which was simultaneously included in qPCR. The primers and methods used for these assays have been reported previously^9^,^54^,^56^.

### Immunofluorescence assay for influenza viral antigen

The infectivity of the influenza viruses in ATI and PMϕ was determined by the percentage of cells that expressed the influenza viral antigens, matrix (M) protein and nucleoprotein (NP). Cell monolayer seeded on coverslips were fixed with 4% PFA for at least one hour. The cell monolayers were washed once with PBS and permeabilized with 0.2% Triton-X-100 for 30 min at RT. Cells were washed with PBS once and stained with a mouse monoclonal antibody conjugated to fluorescein isothiocyanate (FITC) specific to M and NP of influenza A virus (Imagen, USA) for 45 min incubation at 37 °C. The stained cells were washed with PBS once and mounted on glass slides using a mounting medium with 4’,6-Diamidino-2-Phenylindole, Dihydrochloride (DAPI) (Vector Laboratories Inc, USA). Fluorescence images were viewed and captured using a Nikon Eclispe Ti-S inverted microscope system. The percentage of cells with positive influenza viral antigen expression was interpreted as the percentage of infection.

### Control and Statistical analysis

Experiments were performed independently using *ex vivo* cultures from at least three human donors and pigs. ATI and PMϕ were isolated from three human donors. Each virus used for all these experimental replicates was from same batch. MEM inoculated tissue or cells served as mock infection and negative controls.

Results showed in figures were the arithmetic mean and standard error of mean (SEM). The differences between log_10_-transformed viral titers among different viruses at different times post-infection and the gene expression profiles of quantitative cytokine and chemokine of influenza virus–infected cells were compared using two-way ANOVA with a post-hoc *Bonferroni* multiple-comparison test. Differences were considered significant at a *p* value of <0.05. Statistical analysis was performed using Graph-pad Prism 6. As the aim of this part was to determine the effect of PB2 mutations in replication kinetics and cytokine and chemokine induction in human primary ATI and PMϕ, the result generated from the control viruses, H5N1 and H1N1pdm, were only included as reference benchmark.

### Biosafety

All the recombinant viruses with PB2 mutations were loss-of-function mutations. All experiments were carried out in a biosafety level 3 facilities at The University of Hong Kong.

## Additional Information

**How to cite this article**: Chan, L. L. Y. *et al*. Evaluation of the human adaptation of influenza A/H7N9 virus in PB2 protein using human and swine respiratory tract explant cultures. *Sci. Rep.* **6**, 35401; doi: 10.1038/srep35401 (2016).

## Supplementary Material

Supplementary Information

## Figures and Tables

**Table 1:** Influenza viruses used in this study.

Virus	Abbreviation	Subtype	Amino acid residue		
			**PB2**		
			591	627	701
A/California/07/2009	H1N1pdm	H1N1pdm	R	E	D
A/Hong Kong/483/1997	H5N1	H5N1	Q	K	D
Recombinant A/Shanghai/2/2013	rgH7N9	H7N9	Q	K	D
rgH7N9 - PB2 mutants	rgPB2-K627E	H7N9	Q	E	D
	rgPB2-K627E + Q591K		K	E	D
	rgPB2-K627E + D701N		Q	E	N
