# Supplementary material for: Evaluation of the human adaptation of influenza A/H7N9 virus in PB2 protein using human and swine respiratory tract explant cultures
Source: Sci Rep. 2016 Oct 14;6:35401. doi: 10.1038/srep35401 (PMC5064379; doi:10.1038/srep35401)
Supplement: Supplementary Information [file srep35401-s1.pdf]

**Evaluation of the human adaptation of influenza A/H7N9 virus in PB2 protein using human and swine respiratory tract explant cultures**

Louisa LY Chan[1], Christine TH Bui[1], Chris KP Mok[1,2], Mandy MT Ng[1], John M Nicholls[3], JS Malik Peiris[1,2], Michael CW Chan[*1], Renee WY Chan[*1,4]

[1]Centre of Influenza Research and School of Public Health, LKS Faculty of Medicine, The University of Hong Kong, Hong Kong SAR, China; [2]The HKU-Pasteur Research Pole, School of Public Health, LKS Faculty of Medicine, The University of Hong Kong, Hong Kong SAR, China; [3]Department of Pathology, LKS Faculty of Medicine, The University of Hong Kong, Queen Mary Hospital, Hong Kong SAR, China; [4]Department of Paediatrics, Faculty of Medicine, The Chinese University of Hong Kong, Hong Kong SAR, China.

**Supplementary materials**

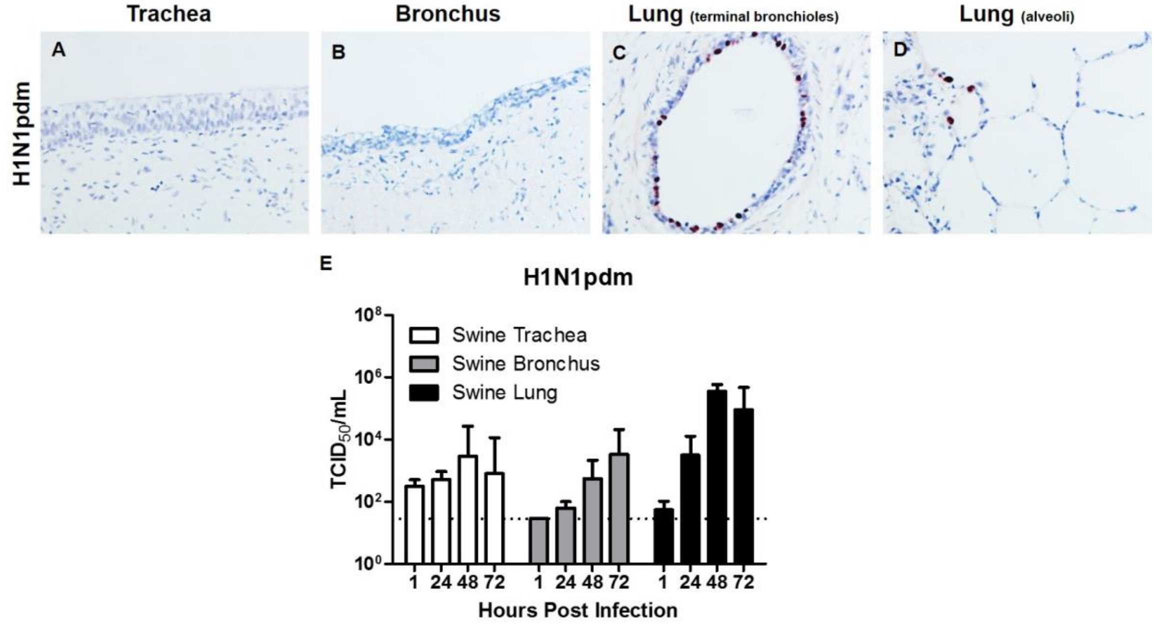


**Supplementary Figure 1.** Viral replication kinetics and Tissue tropism of influenza H1N1pdm in *ex vivo* cultures of swine respiratory organs. Swine trachea, bronchus and lung were infected with $10^6$ $TCID_{50}$ /mL of influenza viruses at 37°C. Formalin-fixed paraffin-embedded sections of swine trachea (A), bronchus (B), terminal bronchioles (C) and alveoli (D) in lung after 24 h infection with H1N1pdm. Sections were stained with a monoclonal antibody against the influenza nucleoprotein with positive cells identified as a red-brown colour and arrows indicated the infected cell. Magnification, × 400. Bar charts show the mean virus titre pooled from at least three independent experiments (E). The horizontal dotted line denotes the limit of detection in the $TCID_{50}$ assay.; error bars show SEM. Key: *$p$ < 0·05, ** $p$ < 0.01, *** $p$ < 0.005.

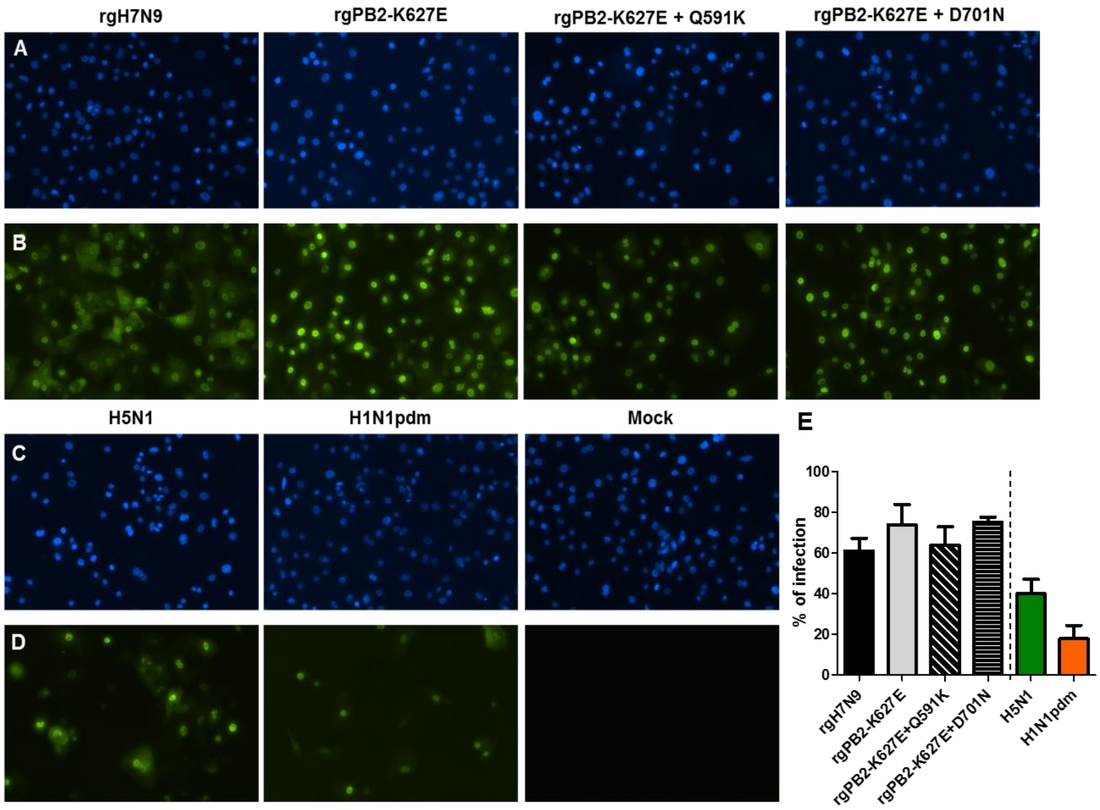


**Supplementary Figure 2.** Immunofluorescence staining of rgH7N9 and its PB2 mutants infected ATI. 4% PFA fixed coverslips seeded with ATI infected with rgH7N9, rgPB2-K627E, rgPB2-K627E + Q591K and rgPB2-K627E + D701N, H5N1 and H1N1pdm were collected at 24 hpi for the staining of influenza nucleoprotein and matrix protein in FITC (green) (B and D) and nucleus in DAPI (blue) (A and C). Magnification x200. Percentage of infection of rgH7N9 and its PB2 mutants in ATI (E). The percentage of infection was indicated as the percentage of the number of cells stained positive for influenza M and NP protein (FITC-green) over the number of cells stained positive for cell nucleus (DAPI-blue). Bars represent the mean and the standard error of mean (SEM) from three sets of independent experiments. No significant differences in the percentage of infection among rgH7N9 and its three mutants were detected using one way ANOVA. The green and orange bars represent the percentage of infection in ATI after the infection of control virus H5N1 and H1N1pdm, respectively.

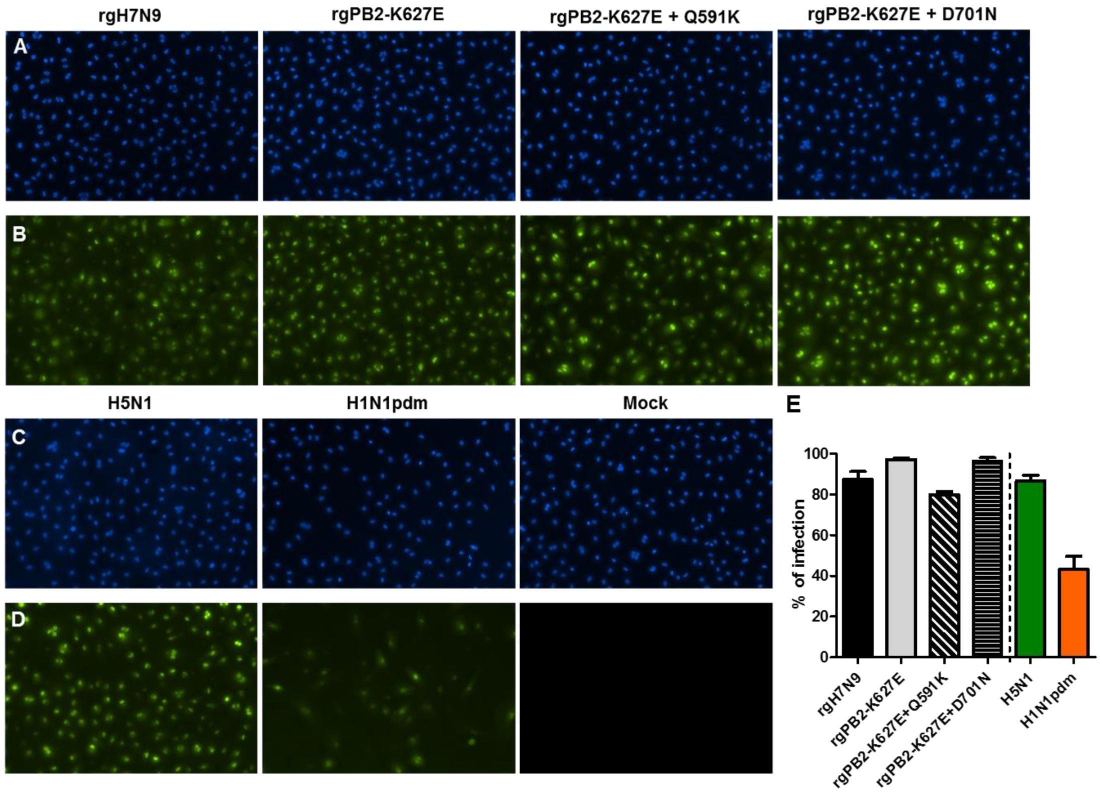


**Supplementary Figure 3.** Immunofluorescence staining of rgH7N9 and its PB2 mutants infected PMϕ. 4% PFA fixed coverslips seeded with ATI infected with rgH7N9, rgPB2-K627E, rgPB2-K627E + Q591K and rgPB2-K627E + D701N, H5N1 and H1N1pdm were collected at 8 hpi for the staining of influenza nucleoprotein and matrix protein in FITC (green) (B and D) and nucleus in DAPI (blue) (A and C). Magnification x200. Percentage of infection of rgH7N9 and its PB2 mutants in PMϕ (E). The percentage of infection was indicated as the percentage of the number of cells stained positive for influenza M and NP protein (FITC-green) over the number of cells stained positive for cell nucleus (DAPI-blue). Bars represent the mean and the standard error of mean (SEM) from three sets of independent experiments. No significant differences in the percentage of infection among rgH7N9 and its three mutants were detected using one way ANOVA. The green and orange bars represent the percentage of infection in PMϕ after the infection of control virus H5N1 and H1N1pdm, respectively.
